# MRI Texture Analysis of Background Parenchymal Enhancement of the Breast

**DOI:** 10.1155/2017/4845909

**Published:** 2017-07-24

**Authors:** Yasuo Amano, Jun Woo, Maki Amano, Fumi Yanagisawa, Hiroshi Yamamoto, Mayumi Tani

**Affiliations:** ^1^Department of Radiology, Nihon University Hospital, 1-6 Kanda-Surugadai, Chiyoda-ku, Tokyo 101-8309, Japan; ^2^Department of Radiology, Juntendo Nerima Hospital, 3-1-10 Nerima-ku, Tokyo 177-8521, Japan; ^3^Division of Radiological Technology, Nihon University Hospital, 1-6 Kanda-Surugadai, Chiyoda-ku, Tokyo 101-8309, Japan; ^4^Department of Breast Surgery, Nihon University Hospital, 1-6 Kanda-Surugadai, Chiyoda-ku, Tokyo 101-8309, Japan

## Abstract

**Purpose:**

The purpose of this study was to determine texture parameters reflecting the background parenchymal enhancement (BPE) of the breast, which were acquired using texture analysis (TA).

**Methods:**

We investigated 52 breasts of the 26 subjects who underwent dynamic contrast-enhanced MRI. One experienced reader scored BPE visually (i.e., minimal, mild, moderate, and marked). TA, including 12 texture parameters, was performed to distinguish the BPE scores quantitatively. Relationships between the visual BPE scores and texture parameters were evaluated using analysis of variance and receiver operating characteristic analysis.

**Results:**

The variance and skewness of signal intensity were useful for differentiating between moderate and mild or minimal BPE or between mild and minimal BPE, respectively, with the cutoff value of 356.7 for variance and that of 0.21 for skewness. Some TA features could be useful for defining breast lesions from the BPE.

**Conclusion:**

TA may be useful for quantifying the BPE of the breast.

## 1. Introduction

Background parenchymal enhancement (BPE) is defined as the initial enhancement of the normal breast tissue in the standardized dynamic contrast-enhanced magnetic resonance imaging (MRI). BPE is categorized as minimal, mild, moderate, and marked according to the Breast Imaging Reporting and Data System (BI-RADS) [1–3]. The factors that influence the degree of BPE are patients' age, vascular supply to the breast tissues, and endogenous hormone [1, 2]. An increased BPE can affect identification of enhancing breast carcinoma, especially that of ductal carcinoma in situ (DCIS) or that showing nonmass enhancement, and interfere with definition of the margin of breast cancer [3, 4]. Recently, there are controversies about the relationship between the degree of BPE and risk for breast cancer [4–7]. Therefore, the accurate assessment of BPE is important for interpreting breast MRI.

BPE is usually scored on a visual inspection [2, 3]. Although dedicated training on breast MRI interpretation improves interobserver agreement for the assessment of BPE, agreement is still moderate [8]. Tagliafico et al. [9] have indicated that their quantitative tool improves BPE assessment. Texture analysis (TA) is a statistical method to analyze the textures of medical images, including signal intensity, its spatial variation, and gray level cooccurrence of the images [10–12]. TA has been already applied to dynamic contrast-enhanced MRI of the breast lesions to differentiate between benign and malignant lesions or characterize breast malignancies [13, 14]. TA is performed not only on dynamic breast MRI but also on MRI of brain tissues, multiple sclerosis, nasopharyngeal carcinoma, and prostate pathologies [10, 15–18]. In addition, TA is applied to CT to evaluate the heterogeneity of renal carcinoma [19]. However, to our knowledge, TA has not been used to quantify BPE on dynamic breast MRI.

Thus, the purpose of this study was to define texture parameters appropriate for evaluating BPE. In particular, we aimed to differentiate between moderate and mild BPE by utilizing TA on the dynamic contrast-enhanced breast MRI.

## 2. Materials and Methods

### 2.1. Patients

Mammogram had shown breast mass, microcalcification, or focal asymmetrical density, and thereafter breast MRI was performed to determine the presence and extent of the lesions in our institution. The present study inclusion criteria were as follows: (1) sufficient image quality on the 1st postcontrast images of MRI, (2) presence of fibroglandular tissues in the breast, (3) no history of biopsy or surgery of the breast, (4) no history of hormonal treatment or chemotherapy, (5) description of visual BPE score according to the BI-RADS on site firstly, (6) an independent reader noted below being able to score BPE based on the BI-RADS later, and (7) a region of interest (ROI) for TA being able to be placed on the BPE. As a consequence, between May 2016 and November 2016, 26 women who underwent breast MRI for identifying or diagnosing breast lesions and whose BPE was scored according to the BI-RADS were included in this retrospective study. The median age was 45.5 years (range, 25–80 years). Histological diagnosis was acquired from 14 of the 26 patients (invasive ductal carcinoma (IDC), 4; fibroadenoma, 4; mastopathy, 4; DCIS, 2). Breast MRI showed no lesions in 10 patients and no histological confirmation was acquired in the remaining 2 patients whose breast MRI had given category 4b for the lesions. The study was approved by the institutional ethics committee, and all patients gave informed consent for dynamic contrast-enhanced breast MRI.

### 2.2. MRI Protocol

All MRI studies were performed using a 1.5 T unit (Achieva, Philips Healthcare, Best, the Netherlands). A phased-array coil was used for signal reception. After T1- and fat-suppressed T2-weighted imaging and diffusion-weighted imaging, transverse dynamic contrast-enhanced MRI was acquired using fat-suppressed 3-dimensional T1-weighted gradient-echo imaging with the following imaging parameters: repetition time: 4.7 ms; echo time: 2.3 ms; flip angle: 10°; in-plane resolution: 1.0 × 1.1 mm^2^; and slice thickness: 2 mm before zero-filling interpolation. A* k*-space segmentation of 26 and sequential phase ordering were used, and the scan time was 63 sec/phase. The images were acquired before contrast, and 1 min, 2 min, and 5 min after the start of 0.1 mmol/kg gadolinium injection at a rate of 2 ml/sec for gadoteridol and 1 ml/sec for gadobutrol.

### 2.3. BPE Assessment

BPE was scored on the 1st postcontrast images by an independent investigator with 25 years of experience in diagnostic radiology, including 12 years of experience in breast MRI. The investigator belonged to an institution different from that performing breast MRI and was blinded to clinical information of all patients.

### 2.4. TA for BPE

The other investigator, who was blinded to the BPE scores, performed TA for the breast parenchyma using MaZda software (version 4.5, Institute of Electronics, Technical University of Lodz, Poland) [10–12]. This is an open-access software for TA developed by the Polish institution, which provides a large amount of texture parameters, including the histogram, gradient, and cooccurrence matrix, which can be related to the medical images placed on the ROI [11]. In the present study, a ROI was placed on the BPE at the slice including the largest breast parenchyma. We acquired 12 texture parameters as follows: the mean, variance, skewness, and kurtosis of signal intensity (SI), the mean, variance, skewness, and kurtosis of gradient, and entropy in the 4 directions (i.e., EnLL, EnLH, EnHL, and EnHH).

### 2.5. Statistical Analysis

Data are presented as mean ± standard deviation. We evaluated the differences in the 12 texture parameters between the 3 BPE scores (i.e., minimal, mild, and moderate), since no patients showed marked BPE in this study. A factorial analysis of variance test was used for the comparison, followed by the post hoc Bonferroni test. *P* < 0.05 was defined as statistically significant. A receiver operating characteristic analysis was used to define the cutoff values of texture parameters to differentiate the moderate and mild BPE or between the mild and minimal BPE. When breast lesions were found, we evaluated their texture parameters.

## 3. Results

Among 52 breasts of the 26 patients, the 36, 11, and 6 breasts showed minimal, mild BPE, and moderate BPE, respectively. There were no patients with marked BPE, possibly because the MRI was performed during the menstrual cycle when BPE might be reduced and because of some of patients' age (i.e., menopause).

Table 1 summarizes the TA data of the BPE. There were significant differences in the variance and skewness of SI, mean and variance of gradient, EnLH, and EnHL between the moderate, mild, and minimal BPE (*P* < 0.01 for all). The variance of SI was significantly greater in the moderate BPE than in the mild and minimal BPE (*P* < 0.01 for both). The variance of gradient and EnLH were also significantly greater in the moderate BPE than in the mild (*P* < 0.05 for both) and minimal BPE (*P* < 0.01 for both). The skewness of SI was significantly greater in the moderate and mild BPE than in the minimal BPE (*P* < 0.01 for both). The mean of gradient and EnHL were significantly greater in the moderate BPE than in the minimal BPE (*P* < 0.05).

**Figure 1:**
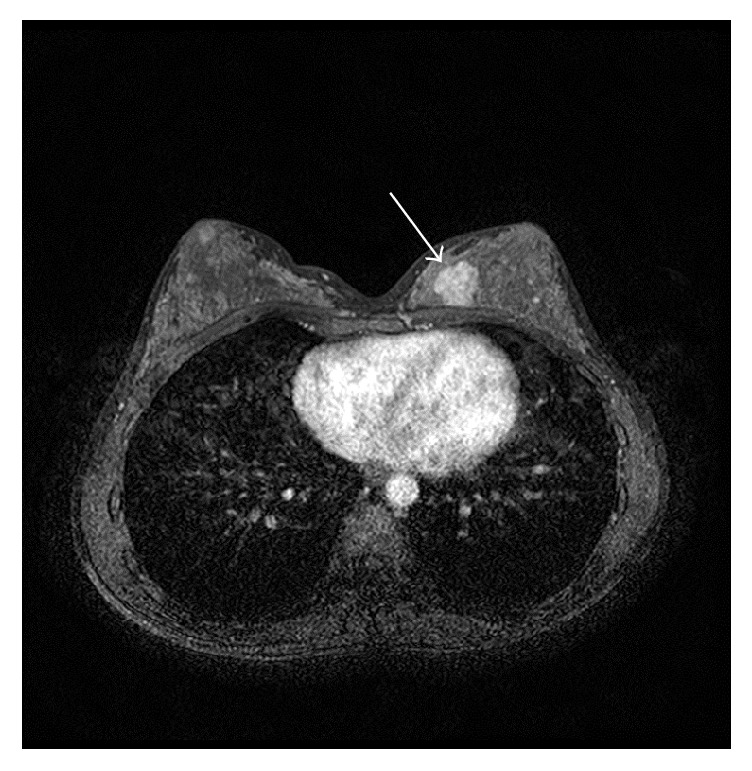
A 32-year-old woman with fibroadenoma. Background parenchymal enhancement (BPE) of the right breast is scored as moderate, while that of the left breast is scored as mild. Texture analysis (TA) reveals that the variance of signal intensity (SI) is 356.7 for the right breast and 310.5 for the left breast. A fibroadenoma is hyperintense to BPE (arrows).

**Figure 2:**
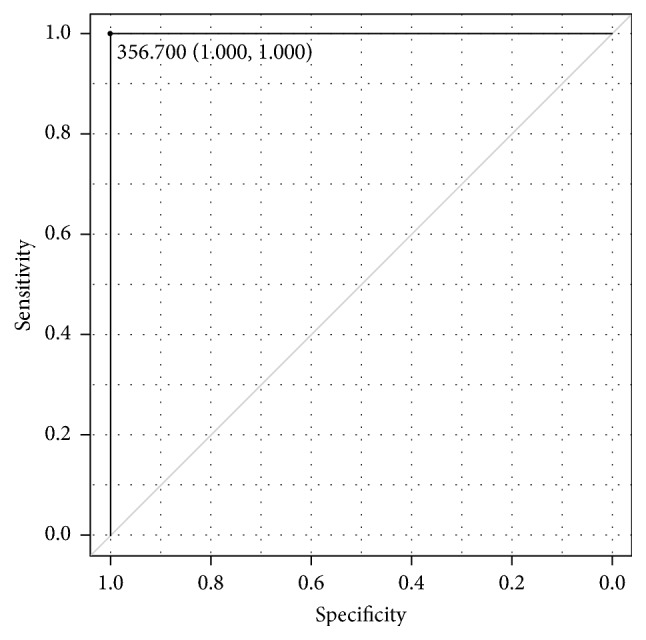
The receiver operating characteristic curve shows that the signal intensity variance of 356.7 is the perfect cutoff value between moderate and mild background parenchymal enhancement.

**Figure 3:**
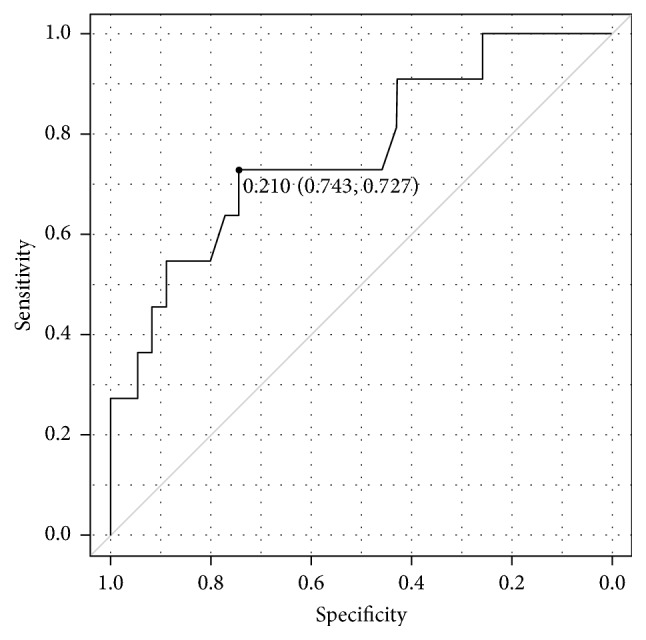
The receiver operating characteristic curve shows that skewness of signal intensity distinguishes between mild and minimal background parenchymal enhancement with the cutoff value of 0.21 and area under the curve of 0.73.

The SI variance of 356.7 was the perfect cutoff value with an area under the curve (AUC) of 1.00 for distinguishing between the moderate and mild BPE (Figures 1 and 2). The cutoff value and AUC for distinguishing between the moderate and mild BPE were 202.8 and 0.82 for the variance of gradient, respectively, and they were 22.8 and 0.79 for the EnLH, respectively. For differentiating between the mild and minimal BPE, the skewness of SI was only a significant texture parameter, and the cutoff value and AUC were 0.21 and 0.73, respectively (Figure 3).

**Figure 4:**
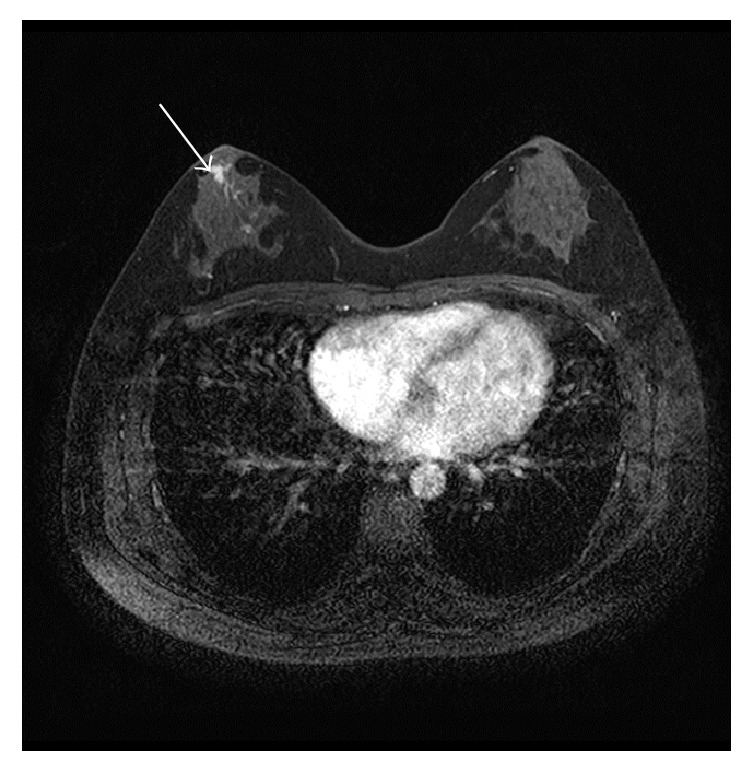
A 41-year-old woman with ductal carcinoma in situ. Background parenchymal enhancement (BPE) is scored as minimal. The tumor (arrow) shows the variance of signal intensity, mean and variance of gradient, and entropy LH and HL higher than their highest values of BPE.

Among the 14 breast lesions proven histologically, the 6 lesions, including 2 DCIS, 2 IDC, and 2 fibroadenoma, were identified at the slices where TA was analyzed (Figures 1 and 4). The 2 IDC and 1 DCIS showed the variance of SI, mean and variance of gradient, EnLL, EnLH, and EnHL higher than their highest values of BPE (Table 2). The 2 fibroadenoma showed the mean SI higher than the highest value of BPE (Table 2). One DCIS was recognized visually, but the texture parameters were within ranges of the parameters of BPE.

## 4. Discussion

TA has been applied to characterize breast lesions and their response to the treatments on dynamic contrast-enhanced MRI [13, 14, 20, 21], whereas TA has not previously been used to quantify BPE. The present study demonstrated that the SI variance acquired by TA provided the perfect cutoff value of 356.7 for distinguishing between the moderate and mild BPE and that the SI skewness was a significant parameter for differentiating between the mild and minimal BPE. Some texture parameters, including the SI variance and EnLL, were higher in 3 of the 4 malignant tumors (i.e., 2 IDC and 1 DCIS) than in BPE. Therefore, the SI variance may be the most useful texture parameter for differentiating between moderate and mild or minimal BPE and between BPE and some malignant lesions.

Strong BPE can interfere with the identification of DCIS [3, 4], and marked or moderate BPE may be one of the risk factors for breast cancer [5, 6]. Thus, quantitative assessment of BPE is required to support the visual scoring of BPE. In the present study using TA, the variance of SI, variance of gradient, and EnLH were significant discriminators between the moderate BPE and the mild or minimal BPE. Among these parameters, the SI variance provided the perfect cutoff value for distinguishing between them. The variance of SI reflects the change ratios of SI between neighbor pixels or within the tissues. Therefore, our results indicate that the visual scoring of BPE can be affected by the heterogeneity and distribution of BPE. The present study also indicates that the SI variance is a useful parameter for identifying IDC [13]. The relationship between entropy and heterogeneity of the breast cancer is likely different between previous studies [20, 21]. The use of subtraction images, TA analysis software, or pathological features of the tumors can affect the values of texture parameters. In the present study, only a skewness of SI was a significant discriminator between the mild and minimal BPE. However, the clinical values of differentiating between them may be small because they are not related to the risks for breast cancer [5, 6].

Previous studies have shown that the semiautomated or automated assessment of BPE correlates with the radiologists' assessment of BPE and can support their visual inspection [9, 22]. The software developed by some authors has been applied to T1- and T2-weighted imaging as well as dynamic contrast-enhanced MRI, but this is home-made [21, 22]. Conversely, the MaZda software used herein is open-access [10–12]. The present study indicates that TA can be applied widely to quantify BPE.

There are several limitations to this study. First, the study population was small. There were a limited number of patients with DCIS. Further study will be requested to determine the usefulness of TA for detecting DCIS surrounded by the moderate BPE. Second, we did not analyze all parameters that could be acquired using TA, because the data handling may be too much to be used clinically. Nonetheless, some texture parameters, including the variance of SI, variance of gradient, and EnLH, were significant discriminators between the moderate BPE and the mild or minimal BPE. Third, TA was performed on only one slice including the largest breast parenchyma, while 3D dynamic contrast-enhanced MRI covered the whole breast. We need a more sophisticated method that can analyze a large amount of TA data rapidly. Fourth, the reference standard was still the BPE scored by an experienced reader visually [8, 9]. Lastly, neither cutoff value nor AUC can be extrapolated to other institutions using 3.0 T or different imaging parameters of the dynamic MRI.

## 5. Conclusions

The variance of SI acquired by TA provided the perfect cutoff value of 356.7 for distinguishing between the moderate and mild BPE in this study. The variance of gradient and EnLH were also significantly greater in the moderate BPE than in the mild BPE. TA can quantify the BPE and help its visual scoring in the clinical practice.

## Figures and Tables

**Table 1:** Texture analysis of background parenchymal enhancement of the breast.

BPE	Minimal (*n* = 35)	Mild (*n* = 11)	Moderate (*n* = 6)	*P *value
*Texture parameters*				
Mean of SI	95.1 ± 23.6	97.4 ± 14.8	101.2 ± 14.0	0.8
Variance of SI	197.8 ± 161.7	273.1 ± 77.3	695.2 ± 236.8	<0.01^*∗*^
Skewness of SI	−0.0.83 ± 0.38	0.33 ± 0.41	0.72 ± 0.47	<0.01^*∗∗∗*^
Kurtosis of SI	0.34 ± 0.62	0.70 ± 1.08	0.71 ± 1.53	0.4
Mean of gradient	14.9 ± 4.69	17.3 ± 2.44	18.2 ± 8.19	<0.01^*∗∗∗∗*^
Variance of gradient	81.1 ± 55.7	99.7 ± 23.7	159.7 ± 65.5	<0.01^*∗∗*^
Skewness of gradient	0.87 ± 0.17	0.91 ± 0.32	0.99 ± 0.32	0.48
Kurtosis of gradient	0.84 ± 0.56	0.92 ± 1.08	1.22 ± 1.16	0.5
EnLL	9452 ± 5199	9775 ± 2997	11326 ± 2967	0.81
EnLH	8.27 ± 4.58	11.46 ± 3.56	17.17 ± 7.10	<0.01^*∗∗*^
EnHL	14.77 ± 10.68	16.76 ± 4.73	24.46 ± 7.85	<0.05^*∗∗∗∗*^
EnHH	2.25 ± 6.77	1.31 ± 0.32	1.62 ± 0.55	0.88

BPE: background parenchymal enhancement, SI: signal intensity, and En: entropy; ^*∗*^the variance of SI was significantly greater in the moderate BPE than in the mild and minimal BPE (*P* < 0.01 for both). ^*∗∗*^The variance of gradient and EnLH were also significantly greater in the moderate BPE than in the mild (*P* < 0.05 for both) and minimal BPE (*P* < 0.01 for both). ^*∗∗∗*^The skewness of SI was significantly greater in the moderate and mild BPE than in the minimal BPE (*P* < 0.01 for both). ^*∗∗∗∗*^The mean of gradient and EnHL were significantly greater in the moderate BPE than in the minimal BPE (*P* < 0.05).

**Table 2:** Texture parameters of the 6 breast lesions.

	Variance of SI	Mean of gradient	Variance of gradient	EnLH	EnHL
IDC^*∗*^	1382.5	35.4	353.5	60.9	52.1
IDC^*∗*^	1656.2	33.8	372.3	44.9	51.4
DCIS^*∗*^	1336.4	33.8	451	45.5	77.1
DCIS	290	18.8	112.1	12.9	15
Fibroadenoma	224.3	16.7	116.8	16.8	26.6
Fibroadenoma	305.8	18.9	170.7	13.9	20.6
Highest value of BPE	685.4	26.3	248.4	22.8	45.7

SI: signal intensity, En: entropy, IDC: invasive ductal carcinoma, DCIS: ductal carcinoma in situ, and BPE: background parenchymal enhancement. ^*∗*^The 2 IDC and 1 DCIS showed the variance of SI, mean and variance of gradient, EnLH, and EnHL higher than their highest values of BPE.

## Abbreviations

BPE: Background parenchymal enhancement
DCIS: Ductal carcinoma in situ
En: Entropy
ROI: Region of interest
TA: Texture analysis.
